# Clinical Reports of Diseases, Injuries, Surgical Operations, Etc., Etc., at the Buffalo Hospital of the Sisters of Charity

**Published:** 1857-11

**Authors:** Benj. H. Lemon

**Affiliations:** Formerly House Surgeon


ART. VI.— *Clinical Reports of Diseases, Injuries, Surgical Operations, &c., &c., in the Male and Female General Surgical and Private Wards of the Buffalo Hospital of the Sisters of Charity.* Care of Prof. HAMILTON, for the winter half year commencing Oct. 1st, 1856. By BENJ. H. LEMON, M. D., formerly House Surgeon.

(Continued from Page 215.)

CASE X.— *Vesico-Vaginal Fistula. Operation.*

Mrs. H., aged 27 years, of Niag. Co., was admitted to a private ward, December 11th, 1856.

She had a vesico-vaginal fistula, produced several years ago in her first labor. It was situated a little back of the neck of the bladder, the longest diameter transversely; it was about an inch in length, and half an inch in breadth.

The urine constantly dribbled away, and she suffered extremely from excoriations.

The operation consisted in freely paring the edges of the fistula, by means of a pair of long forceps and a knife, and then introducing three strong silk sutures.

The operation was made without the aid of anæsthetics, and with the patient lying on her *back*.

The instruments employed were constructed for the purpose, with long handles. In carrying the sutures, the longinversed needles were found especially useful, they were inserted a quarter of an inch or more from the edge of the opening.

The patient was kept on her *belly* for several days after the operation, the urine being drawn away by a metallic catheter kept constantly in the bladder.

The sutures were removed at various periods, but none of them within the first week.

The operation has proved perfectly successful so far as the closure of the fistula is concerned, but she has still some incontinence of urine.

Dr. Hamilton believes that the common interrupted suture, which he has always employed in these operations, is equal to any other form of suture, and that success depends, mainly, upon attention to three points:

*First.* The free incision, or paring of the edges of the fistula.

*Second.* The introduction of the sutures remote from the edges, plunging the needle deep into the tissues, taking care, however, not to include the vesicle mucous membrane, so as to bring a large amount of raw surface into coaptation.

*Third.* The adoption of the *prone* posture after the operation has been completed, to be persevered in for several days, the urine being constantly drained off by the catheter, thus preventing it coming in contact with the wound.

Case XI.— *Organic Stricture of a portion of the Membranous and Bulbous Tract of the Urethra, impregnable to any instrument. Remedy by external incision.*

Henry Compton, aged 28 years. Admitted Jan'y 12, '57.

This patient suffered much from an irritable bladder and urethra, since the age of fourteen. The difficulty constantly increased, and for two years he has been unable to void his urine in a full stream, the great irritability of the bladder and canal causing him to make the attempt many times during the day and night, and often not more than an ounce at a time was evacuated; and at other times, a little guttatim, any imprudence in diet greatly aggravated the difficulty. He was incapable of engaging in any employment, and his life rendered miserable, the greater part of his time being occupied in voiding his urine, his general health also becoming much impaired from sleepless nights and constant pain. He had never been treated by any surgeon.

He remained in the house a month, during which time repeated attempts were made to pass various sized steel sounds through the stricture, at intervals of several days, whilst under the influence of anæsthetics, but at no time could any instrument be got to pass further, with a warrantable degree of force, than just anterior to the belly.

Alkaline and mucilaginous drinks were prescribed.

Prof. Hamilton finally decided to operate by "external incision."

Feb. 16th, surgical clinic day, the patient was placed in the position for lithotomy, and chloroform administered. It excited him at first, and he struggled and cried out, but soon fell into a deep sleep. A steel sound was then passed down the urethra to the point of obstruction. Dr. Hamilton then having introduced the index finger of his left hand into the rectum, cut down on the perineal tissues, including the bulb, to the beak of the sound, one of the arteries of the bulb was cut, the hæmorrhage was considerable, and the artery was tied.

The strictured canal was then sought for, and after much difficulty, found; it was scarcely larger than pack-thread, occupying about four or five lines of the membraneous, and an equal extent of the bulbous tract of the urethra being an "elongated, annular stricture."

The tissues were divided as far as the narrowed canal extended, and the sound passed into the bladder; it was then withdrawn, and a No. 10 silver catheter introduced, and tapes attached to retain it in a proper position.

I visited him early the next morning, and found him in a severe chill, the teeth chattering, and the whole body trembling violently, and groaning like one in the cold stage of an intermittent fever; the paroxysm had continued ten minutes; he had had one in the night which had lasted fifteen minutes.

Directed hot brandy, and a warm blanket.

On examination it was found, that through change of posture, the catheter had become displaced, and the tapes were pulling it to one side and forcing it in in a manner to cause the beak of the instrument to press on the internal coat of the bladder with considerable force; this was, in all probability, the cause of the chills. As soon as the catheter was adjusted the chill ceased.

The urine flowed from the end of the catheter—none came through the wound.

A cork was placed in the end of the catheter, and the dresser directed to remove it at intervals, and keep the instrument carefully adjusted.

Third morning. He had no chills since yesterday; on removlng the cork the urine flowed; the wound was in good condition, and no urine had passed through it.

The patient continued to do well up to the close of Dr. Hamilton's service at the end of February; the wound healed kindly, and I understand that no water ever passed through it.

The catheter, however, became obstructed at one time. with mucus and pus; to avoid the risk of great difficulty, and to spare the patient the pain of returning the iustrument, should it be withdrawn, Dr. Hamilton applied his mouth to the end of it, until the obstructing substances were removed.

I saw the man after his discharge from the hospital at the end of near three and a-half months after the operation; his general health is greatly improved, and he can void his urine in *a full stream*, to the distance of several feet, and suffers no inconvenience, except from an irritable bladder, which causes a desire to urinate frequently: he is slowly improving in this respect.

There is yet a ligature to come away. I advised a bullet to be attached to it, to facilitate the process, or an elastic string, attached to the thigh by adhesive plaster.

The virile functions are unimpaired.

Mr. Syme states, in a paper to the Royal Medical and Chirurgical Society, "that whenever urine can pass through the urethra, an instrument may be made to do so, not perhaps at once, and with ease, but *always* through time and proper management."

Other eminent surgeons, who were present, Travers, Hutchison, and Coulson, did not concur in this view.

It is, indeed, difficult, either in medicine or surgery, to lay down any general rule that will not be frequently liable to exceptions, as the foregoing case is an exception to that of Mr. Syme.

There was an "elongated annular stricture," the urethral canal almost obliterated by the thickening and condensating of the tissues, occupying parts of the bulbous and membranous tracts of the passage, complicated by the pouch or "cul-de-sac" at the bulb, the triangular ligament, and the angle which the urethra here takes.

